# Insights into aluminum-tolerance pathways in *Stylosanthes* as revealed by RNA-Seq analysis

**DOI:** 10.1038/s41598-018-24536-3

**Published:** 2018-04-17

**Authors:** Caode Jiang, Lusheng Liu, Xiaofeng Li, Rongrong Han, Yunmin Wei, Yongxiong Yu

**Affiliations:** 1 Forage Laboratory, College of Animal Science and Technology, Southwest University, Chongqing, 400715 China; 2 College of Agriculture, Guangxi University, Nanning, 530005 China

## Abstract

Stylo has a great potential for Al^3+^ resistance in acidic soils through secretion of citrate from the roots. To get insight into the molecular mechanisms responsible, transcriptomic changes were investigated in the roots after treatment with T01 (−Al^3+^, pH6.0), T02 (−Al^3+^, pH4.3) and T03 (50 µM AlCl_3_, pH4.3). In total, 83,197 unigenes generated from 130,933 contigs were obtained. Of them, 282, 148 and 816 differentially expressed unigenes (DEGs) were revealed in T01_vs_T02, T02_vs_T03 and T01_vs_T03 comparison, respectively (FDR < 0.001, log_2_FC > 2). DEGs by Al^3+^ were related to G-proteins, diacyglycerol and inositol metabolism, calcium-signaling, transcription regulation, protein modification and transporters for detoxification of Al^3+^. Additionally, Al^3+^ facilitates citrate synthesis via modifying gene expression of pathways responsible for citrate metabolism. Overall, Al^3+^ resistance in stylo involves multiple strategies and enhancement of citrate anabolism. The Al^3+^ signal transmits through heterotrimeric G-proteins, phospholipase C, inositol triphosphate, diacylglycerol, Ca^2+^ and protein kinases, thereby activating transcription and anion channels in plasma membrane, and resulting in citrate secretion from stylo roots.

## Introduction

Trivalent aluminum (Al^3+^) toxicity is a major constraint for root growth and crop yields of plants in acidic soils, which constitute 50% of the potentially arable lands worldwide^1^. At soil pH below 5.0, the soluble Al^3+^ species damages cells at the root apex, and subsequently inhibits the uptake of water and nutrient^2,3^. Although Al^3+^ toxicity can be ameliorated through application of lime which raises the soil pH, this amendment does not reduce acidity in the subsoil layer. Moreover, liming is not a cost effective method, thus may not always be practical. Alternatively, one of the most appropriate strategies to increase crop productivity on acidic soils is to cultivate Al^3+^ resistant varieties. A solid understanding of Al^3+^-resistance mechanisms will facilitate development of improved crops well suitable for acidic soils.

Plants thriving in acidic soils have evolved both external avoidance and internal tolerance mechanisms. The external avoidance mechanisms prevent Al^3+^ from entering root cells by secretion of organic acid anions (OAs), such as citrate, malate and oxalate, from the root apex, resulting in the formation of stable nonphytotoxic OA-Al chelates^2,3^. The internal tolerance mechanisms enable plants to uptake and sequester Al^3+^ in the root vacuole once it enters the cell in the cytosol, or to allow the plant to tolerate the Al^3+^ in the cell wall.

Over the past decades, great efforts have been made to identify the genes for external and internal detoxification of Al^3+^ in plants. The first class of resistance genes encoding malate and citrate efflux transporters (ALMTs) has been identified in wheat, *Arabidopsis*, rape and rye^2^. Another protein family involved in Al^3+^ tolerance is from multidrug and toxic compound extrusion proteins (MATEs) that are associated with Al^3+^-activated citrate exudation in plant species^2^. Internally, Nramp aluminum transporter 1 (Nrat1), operates in concert with a tonoplast-localized half-size ABC transporter, ALS1, to remove Al^3+^ from the cell wall, and to sequester it in the root-cell vacuole in rice^3,4^. Subsequent studies have identified sensitive to proton rhizotoxicity 1 (STOP1) in *Arabidopsis* and Al^3+^ resistance transcription factor 1 (ART1) in rice as transcription factors that regulate expression of a suite of Al^3+^-resistance genes^5,6^. Despite of these findings, the mechanisms that modulate gene expression responsible for Al^3+^-induced secretion of citrate remain unclear.

Stylo (*Stylosanthes*) has a great potential for Al^3+^ tolerance on highly acidic soils prevalent in the tropics and sub-tropics. We documented a cultivar genotype Reyan 2 (R2) of stylo exhibiting high Al^3+^ resistance via Al^3+^-induced release of citrate from root apex^7^. In addition, citrate efflux upon Al^3+^ exposure was delayed by several hours, suggesting that Al^3+^ induces the expression of responsive genes in this species. Yet, to date, research on mechanisms of stylo responses and tolerance to Al^3+^ have been hampered because of the lack of the genomic sequence and transcriptomic data under Al^3+^ stress. Here, the RNA-Seq was applied to analyze the transcriptomic changes in stylo roots after Al^3+^ exposure. The aim was to characterize the pathways that orchestrate expression of genes responsible for Al^3+^-induced secretion of citrate in stylo.

## Results

### Optimal concentration and duration of Al^3+^ treatment

**Figure 1:**
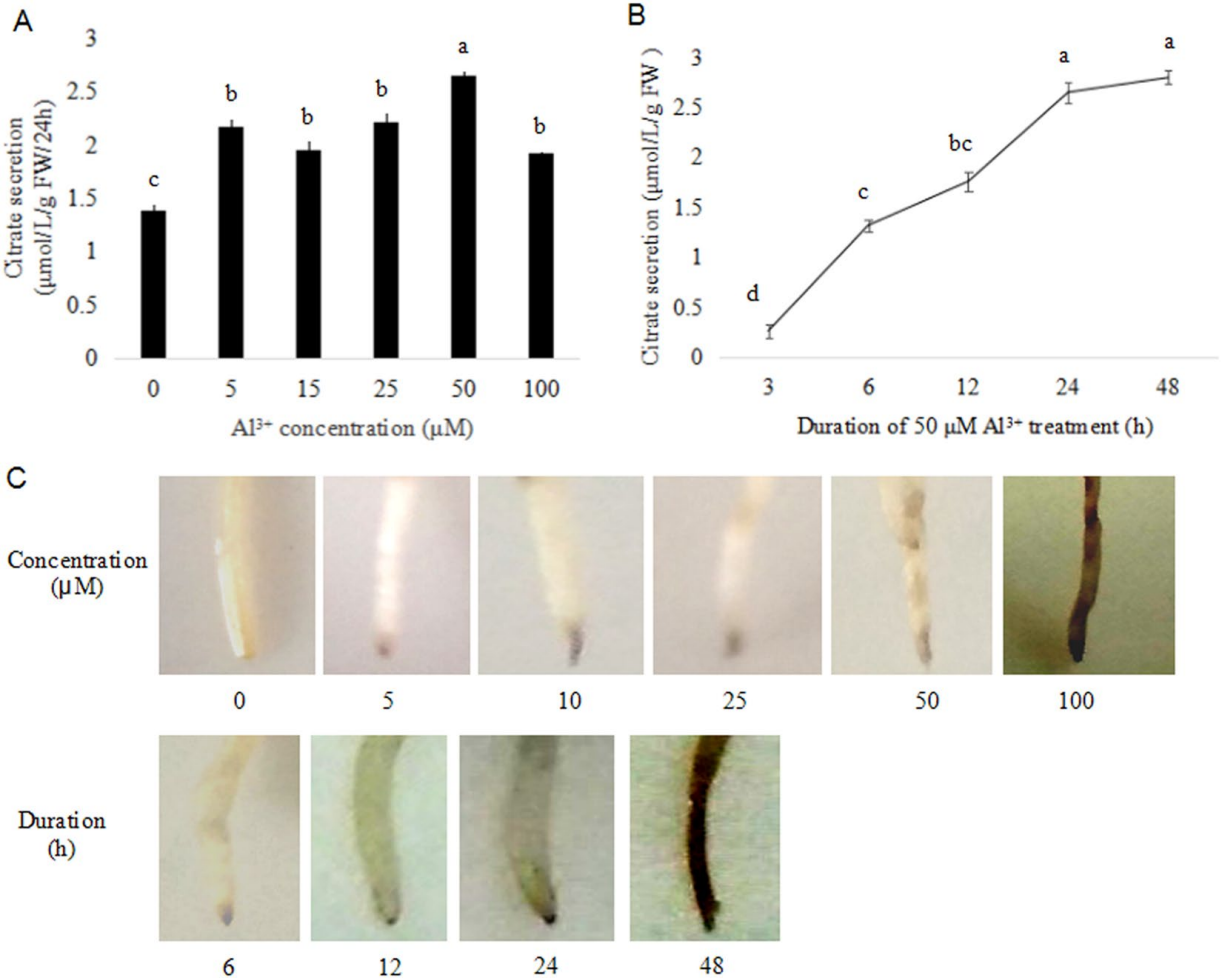
Effect of AlCl_3_ concentration for 24 h (**A**) and duration of 50 µM AlCl_3_ treatment (**B**) on citrate secretion in R2 roots and Chrome Azurol S staining (**C**). Values are represent as Mean ± SE (n = 5). Different letters indicate significance at *P* < 0.05.

We previously reported that in R2 roots the amount of citrate efflux was Al^3+^ concentration and time dependent^7^. To further optimize Al^3+^ treatment for RNA-Seq analysis, measurement of citrate secretion was performed at different dosage and treatment time of Al^3+^. The largest quantity of citrate exudation was detected at 24 h after 50 μM AlCl_3_ (pH4.3) treatment with no more than a 25% inhibition of relative root growth (RRG) compared to the control (−Al^3+^) (Fig. 1A,B). Although Chrome Azurol S staining deepened with increasing of Al^3+^ concentration and treatment time, the 24 h group demonstrated much less damage in the roots than that of the 48 h group (Fig. 1C).

### Transcripts *de novo* assembly and annotation

RNA-Seq of T01 (control, −Al^3+^, pH6.0), T02 (acid treatment, −Al^3+^, pH4.3) and T03 (+Al^3+^, 50 µM AlCl_3_, pH4.3) treatments generated a range of 20.59–34.36 million clean reads per sample (Table 1), which represented an range of 91.3% to 93% of the raw reads. In total, there were 61.91 Gb clean paired-end reads from all samples with a GC content of about 45% and with equal distribution of G and C (or A and T) in the reads. *De novo* assembly of the reads produced 130,933 transcripts corresponding to 83,197 unigenes (Table S1). The assembled unigenes had a length distribution from 203 to 4,017 bp with an N50 length of 1,307 bp and an average length of 959 bp (Table S1), and 54,976 unigenes were annotated (Table S2). Saturation simulation for transcriptomic data showed that the number of novel genes decreased with total read number increasing in each sample (Fig. S1), and a good correlation between the replicates was observed (R^2^ > 0.847, Fig. S2A).

**Table 1:** Summary of short-read data of RNA-Seq.

Sample	Treatment	Clean Read	GC (%)	≥Q30 (%)	Mapped Read	Expressed Unigene
T01–1	T01(−Al^3+^, pH6.0)	23,593,254	45.41	92.50	19,028,551	52,176
T01–2		20,594,687	45.26	91.93	14,329,659	65,806
T01–3		23,598,964	45.25	92.01	17,737,584	62,682
T02–1	T02(−Al^3+^, pH4.3)	23,359,649	45.09	92.71	19,325,346	48,568
T02–2		34,364,939	44.58	92.20	26,577,280	60,741
T02–2		21,974,723	45.21	92.32	16,229,807	60,414
T03–1	T03(+Al^3+^, pH4.3)	24,804,328	45.17	92.79	20,331,769	49,133
T03–2		22,992,711	44.85	92.60	16,830,708	61,540
T03–3		23,474,652	44.97	92.01	17,067,386	60,030

### Global transcriptomic analysis of Al^3+^-affected genes

Differentially expressed unigenes (DEGs) in R2 roots between treatments were further analyzed. A total of 281 DEGs were discovered in the comparison of T01_vs_T02 (2 up-regulated and 279 down-regulated), whereas there were 148 DEGs in T02_vs_T03 with 49 increasing and 99 decreasing in expression (FDR < 0.001, log_2_FC (fold change) > 2) (Table 2). The number of DEGs in T01_vs_T03 was 819, of which 40 unigenes were induced and 799 unigenes were inhibited. A hierarchical clustering and heatmap analysis showed that the expression patterns observed in plants of acid and Al^3+^ treatment clustered together, which showed clear difference to the control plants (Fig. S2B).

**Table 2:** Differentially expressed unigenes between treatments.

Group	Total	Up-regulated	Down-regulated
T01_vs_T02	281	2	279
T02_vs_T03	148	49	99
T01_vs_T03	819	40	799

### Functional classification of DEGs

**Figure 2:**
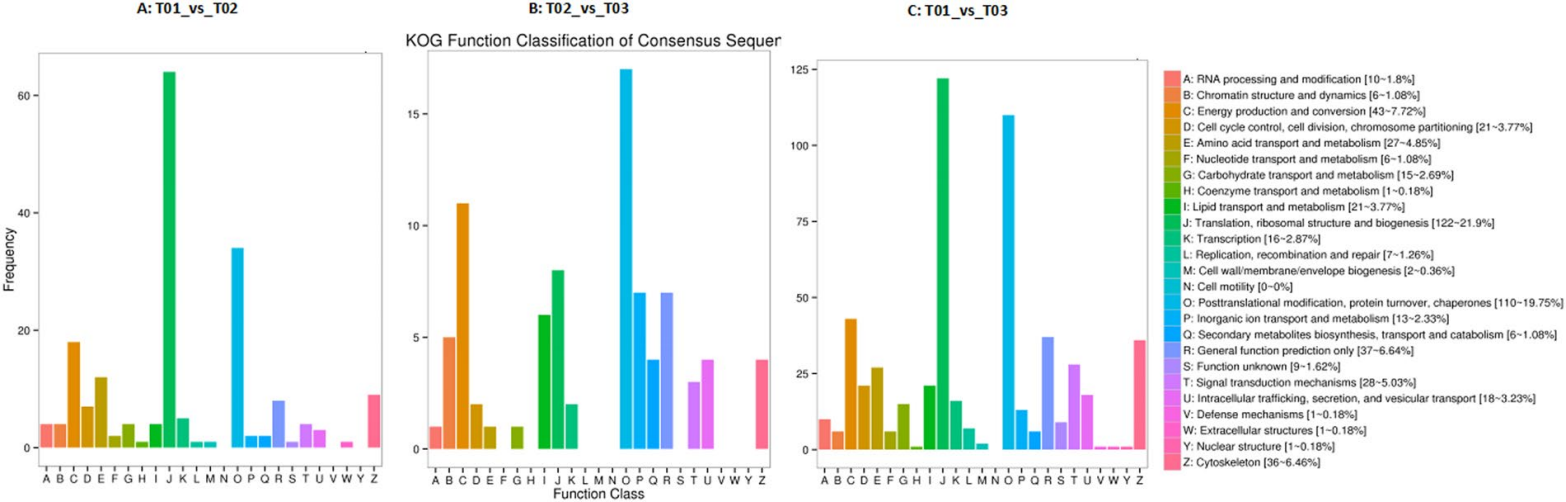
Histogram of KOG classification for differentially expressed genes in R2 roots.

The annotated DEGs were compared with Eukaryotic Orthologous Groups (KOG) database for functional prediction and the putative proteins were classified into 25 KOG groups. In T01_vs_T02, the larger groups included “translation, ribosomal structure and biogenesis”, “posttranslational modification, protein turnover, chaperones”, “energy production and conversion”, “amino acid transport and metabolism” and “cytoskeleton” (Fig. 2). This result was supported by T02_vs_T03 and T01_vs_T03 except “inorganic ion transport and metabolism” and “signal transduction mechanisms” instead of “cytoskeleton”, respectively. GO (Gene Ontology) enrichment on the basis of the biological process (BP), cellular component (CC) and molecular function (MF) showed that most affected GO terms common in the three groups included “translation”, “regulation of transcription, DNA-templated” and “small GTPase mediated signal transduction” in BP, “ribosome” and “small ribosomal subunit” in CC, and “structural constituent of ribosome”, “beta-galactosidase activity” and “phospholipase activity” in MF (Table S3). GO terms only represent in T01_vs_T03 included “regulation of response to biotic stimulus”, “regulation of response to external stimulus” and “xylan metabolic process” in BP, “trans-Golgi network”, “organelle” and “extracellular region” in CC, and “transition metal ion binding” in MF (Table S3). For Kyoto Encyclopedia of Genes and Genomes (KEGG) pathway enrichment analysis, DEGs related to “ribosome”, “nitrogen metabolism” and “citrate cycle” were significantly enriched in both T02_vs_T03 and T01_vs_T03 groups, but “aminoacyl-tRNA biosynthesis”, “fatty acid degradation” and “tryptophan metabolism” were only over-represented in T01_vs_T03 group (*P* < 0.05, rich factor > 2, Fig. S3).

### Al^3+^-affected transporter genes in R2 roots

Transporters are required for Al^3+^-induced secretion of OAs and for Al^3+^ uptake, sequestration and distribution^8^. Therefore, we extracted transporter genes whose expression levels were altered by Al^3+^. In total, 36 transporter genes were found (Table 3, Table S4). Among them, 16 unigenes were up-regulated and 20 unigenes were down-regulated. The upregulated genes belonged to ATP-binding cassette (ABC) transporter family, multidrug and toxic compound extrusion (MATE) family, major facilitator superfamily (MFS), inorganic ion transporters, H^+^-ATPase, lipid transport and metabolism and others.

**Table 3:** Summary of unigenes affected by Al^3+^.

Gene category	Total No.	Up-regulated	Down-regulated
Transporter	36	16	20
Transcription	16	2	14
Translation and posttranslational modification	4	4	0
Signal transduction	37	6	31

### Influence of Al^3+^ on the genes involved in citrate metabolism

**Figure 3:**
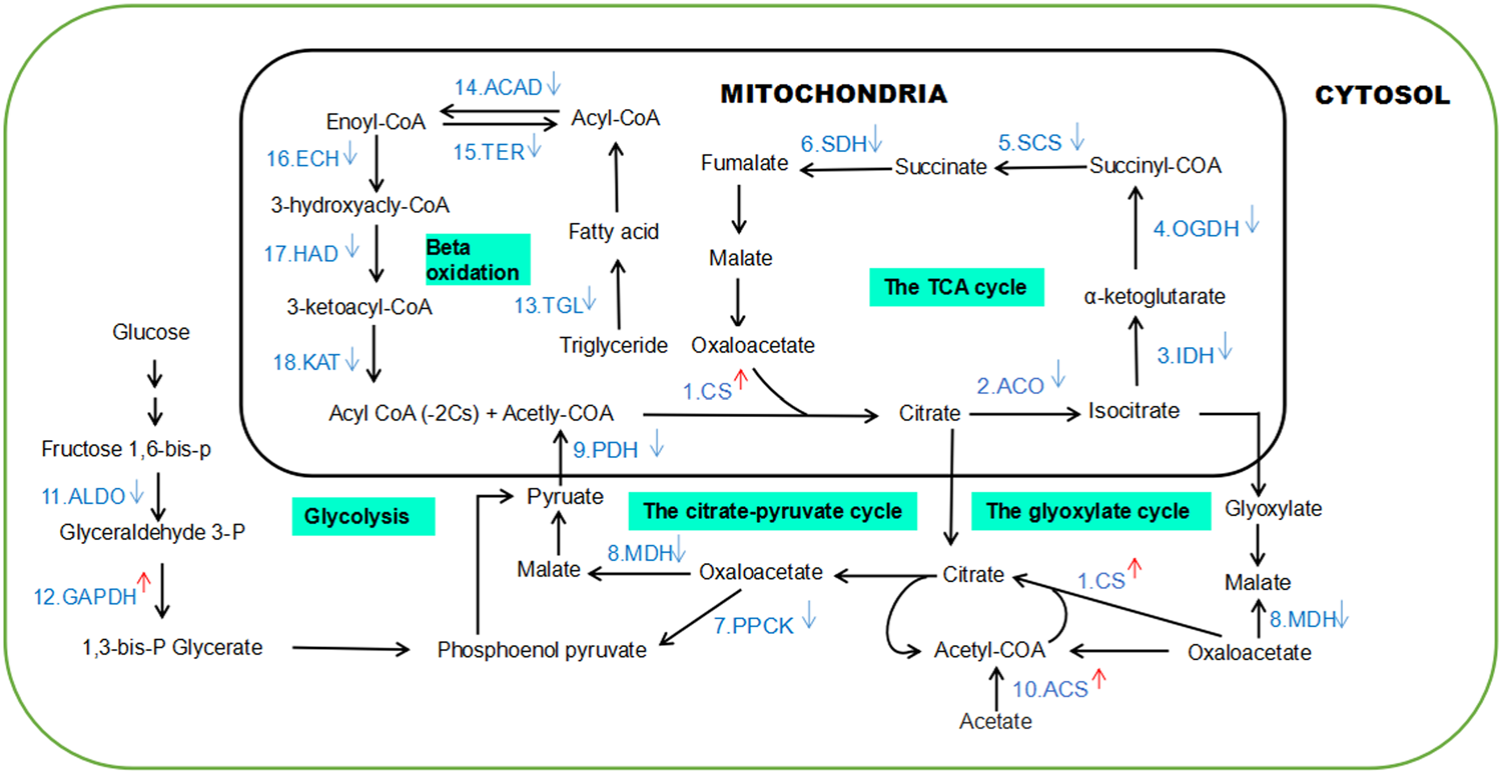
Expression changes of genes involved in citrate metabolism under Al^3+^ treatment. 1. CS, citrate synthase; 2. ACO, aconitate hydratase; 3. IDH, isocitrate dehydrogenase; 4. OGDH, 2-oxoglutarate dehydrogenase; 5. SCS, succinyl- CoA synthetase; 6. SDH, succinate dehydrogenase; 7. PPCK, phosphoenolpyruvate carboxykinase; 8. MDH, malate dehydrogenase, cytoplasmic; 9. PDH, pyruvate dehydrogenase; 10. ACS, acetyl-coenzyme A synthetase, chloroplastic/glyoxysomal; 11. ALDO, fructose-bisphosphate aldolase; 12. GAPDH, glyceraldehyde-3-phosphate dehydrogenase; 13. TGL, triacylglycerol lipase; 14. ACAD, acyl-CoA dehydrogenase; 15. TER, trans-2-enoyl-CoA reductase, mitochondrial; 16. ECH, enoyl-CoA hydratase; 17. HAD, 3-hydroxyacyl-CoA dehydrogenase; 18. KAT2, 3-ketoacyl-CoA thiolase 2. Significant difference between treatments is indicated with ^↑^ (increase in expression) and ^↓^ (decrease in expression).

As citrate play an important role in Al^3+^ tolerance in R2 roots^7^, we examined the genes associated with citrate metabolism. For genes in tricarboxylic cycle (TCA), CS experienced a more than 4-fold increased expression by Al^3+^, but transcription levels of ACO, IDH, OGDH, DLST, SCS and SDH were decreased (Fig. 3, Table S5). No change of gene expression in the glyoxylate cycle was observed except elevated transcripts of ACS and CS, which catalyze acetate to acetyl-COA, then to citrate. As regards the genes in glycolysis pathway, GAPDH was up-regulated though ALDO and PPCK were down-regulated. Of interest, decreased expression was observed for genes related to triglyceride metabolism (TGL) and fatty acid beta-oxidation (HCAD, TER, ECH, HAD and KAT), and down-regulation of MDH and PDH in citrate-pyruvate cycle was also detected.

### Expression changes of genes associated with transcription and translation

Twenty unigenes, mainly encoding transcription factors (9) and translation and posttranslational modification proteins (4), were affected with Al^3+^ treatment (Table 3, Table S6). Particularly, Al^3+^ enhanced the expression of transcription factor STOP1 and unigenes involved in translation and posttranslational modification. Previous reports have determined that STOP1/ART1 regulates 32 genes in rice and 43 genes in *Arabidopsis*^2,5^. In R2 roots, Al^3+^ altered 6 STOP1-regulated genes in expression, of which 3 unigenes were down-regulated, and 4 unigenes, which belong to ALS1 homolog, MATE family and DUF642 family, were up-regulated (Table S7).

### Expression changes of genes associated with signal transduction

Six unigenes involved in cell signal were increased while 31 unigenes were decreased in expression by Al^3+^ (Table 3, Table S8). These unigenes were associated with ras family and G-proteins (4), diacyglycerol and inositol metabolism (2), calcium-binding proteins (4), CBL-interacting protein kinases (CIPK) (3), serine/threonine kinases (10), Carboxylesterase (3), phosphatases (4) and others (6).

### Validation of RNA-Seq data by qRT-PCR

To validate the results of RNA-Seq, real-time RT-PCRs were performed for 7 DEGs (PIPK5D6, GPA2, PLC, INPS1, DGAT, STOP1, ABCC9) involving the G-protein signaling pathways and citrate secretion (Table S9). A significant correlation (R^2^ = 0.89, *P* < 0.05) was observed between the two data sets.

### Pharmacological disclosure of Al^3+^-signaling pathways

**Figure 4:**
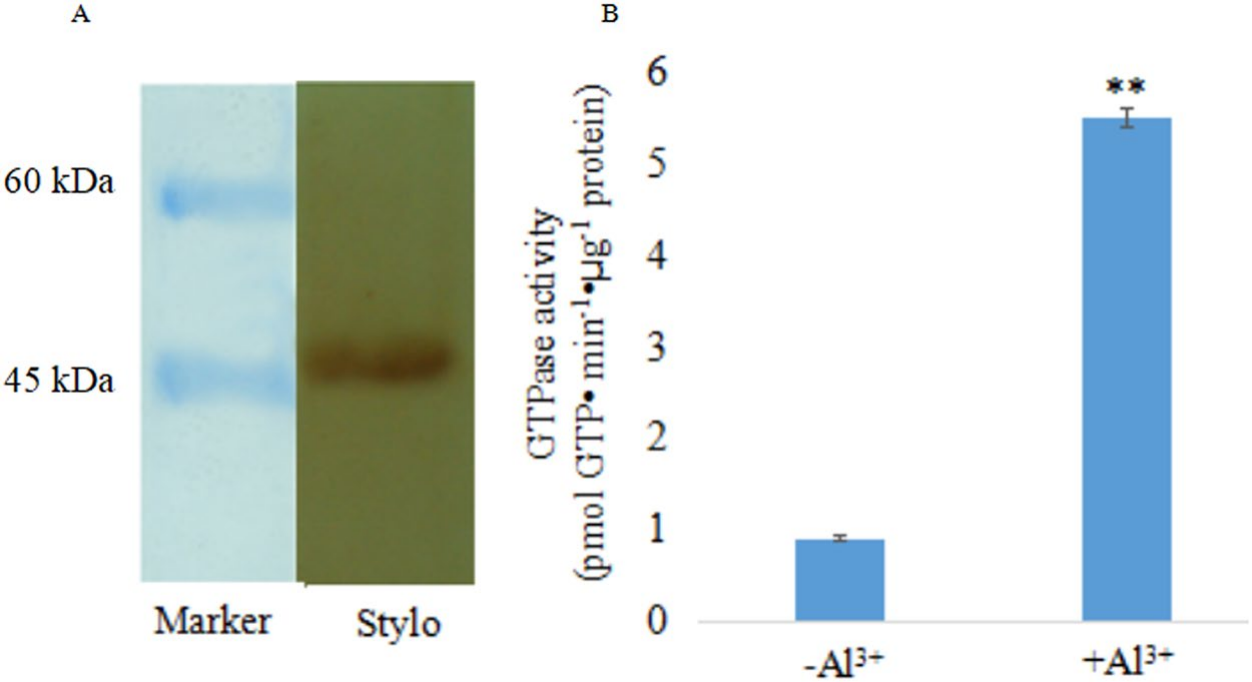
Effect of Al^3+^ on the GTPase activity in the plasma membrane of R2 roots. (**A**) Immunoblot analyses for heterotrimeric G-proteins probed with antibody raised against the *Arabidopsis* Gα. (**B**) R2 seedlings were exposed to 0.5 mM CaCl_2_ solution containing 0 (−Al^3+^) or 50 μM AlCl_3_ (+Al^3+^) at pH 4.3 for 3 h. Vertical bars represent Mean ± SEM (n = 3). Pairwise Student’s t test was used to compare values to the control (−Al^3+^). **Significant at *P* < 0.01.

Our early investigation detected the activation of GTPase in response to Al^3+^ in root plasma membrane of *Arabidopsis* and rye^9^. To test the role of heterotrimeric G-proteins in Al^3+^-induced secretion of citrate in stylo roots, presence of the proteins in the plasma membrane of R2 roots was evaluated (Fig. 4). After exposure to 50 μM AlCl_3_ (+Al^3+^) for 3 h, GTPase activity in root plasma membrane was elevated about 5 times compared to that of the control (−Al^3+^) (*P* < 0.01, Fig. 4). Citrate secretion from R2 roots was significantly stimulated by G-proteins agonist cholera toxin (CTX, 5–50 ng/mL), whereas the secretion was significantly inhibited by the antagonist pertussis toxin (PTX, 50–200 ng/mL) in a dose-dependent manner (*P* < 0.05) (Fig. S4A). In the absence of Al^3+^, citrate secretion from roots was not detectable, regardless of whether the roots were exposed to CTX or not. To support the involvement of G-proteins in Al^3+^ enhancement of citrate excretion, the downstream effectors were also analyzed pharmacologically. Citrate exudation was significantly induced (*P* < 0.05) by the specific agonist of IP_3_ receptor thapsigargin (TSG, 2–10 µM), but was inhibited by the specific antagonsits of phosphoinositide phospholipase C (PLC) (neomycin, 5–20 µM and U73122, 2–8 µM), inositol trisphosphate (IP_3)_ receptor (2-aminoeyhyl diphenylborinate (2-APB), 5–20 µM), diacylglycerol (DAG) (R599499, 2–8 µM) and protein kinase (K-252a, 1–10 µM) (Fig. S4B–S4E). Also, CTX stimulated exudation of citrate under Al^3+^ treatment was arrested by anion channel antagonsits phenylglyoxal (PG) and anthracene-9-carboxylic acid (A-9-C). After co-treated CTX (20 ng·mL^−1^) with PG and A-9-C (30 µmol·L^−1^), secretion rate of citrate dropped to 71.4% (CTX + PG) and 72.0% (CTX + A-9-C), respectively (*P* < 0.05, Fig. S4F).

## Discussion

In the present work, the optimal concentration and duration of Al^3+^ treatment, at which citrate secretion reached its highest level with slight damage in the roots, was determined for RNA-Seq analysis. This makes it possible to analyse Al^3+^-tolerance genes in stylo. The high Q-score and no GC and AT separation in clean reads indicate the high accuracy of base calling in RNA-Seq data (Table 1, Fig. S1). Especially, the N50 length of transcripts and unigenes (Table S1) and the number of genes detected with mapped reads tending to stabilize in each sample (Fig. S1) suggest the high integrity of assembly. Thus, the transcriptome assembly provides a rich source for investigating the genes and pathways related to Al^3+^-tolerance in stylo.

The differential expression analysis identified that most of DEGs were down-regulated in acidic Al^3+^ treatment (Table 2). These results are consistent with the fact that stylo thrives in acidic soil but not in neutral environment in practice, reflecting the adaptation of stylo to acidic Al^3+^ soil but being stressed under neutral environment. KOG and GO classification and KEGG pathways of DEGs enriched in acid and Al^3+^ treatment were involved in regulation of transcription, ribosome, translation and posttranslational modification (Figs 2 and S3). Especially, Al^3+^ enhanced the expression of transcription factor STOP1, ribosomal protein S30 and 3 other unigenes functioning in posttranslational modification (Table S6). These results lend supports to the observation that the protein-synthesis inhibitor, cycloheximide, significantly inhibits Al^3+^-induced secretion of citrate^7^, and emphasize the importance of transcription and translation regulation and protein modification during the activation of Al^3+^resistance responses in stylo.

Of particular interest, DEGs associated with transporters were over-represented. The importance of membrane-localized MATE transporters and H^+^-ATPase in mediating OA exudation has been well documented^10^. We also established the important role of anion-channel or citrate transporters in Al^3+^-enhancement of citrate secretion in stylo roots using pharmacological methods^7^. In this work, although only 2 unigenes induced by Al^3+^ were annotated as MATE transporter family (Table S4), high expression levels of MATE efflux family protein FRD3 and NRAMP family Nramp5 were detected. Al^3+^-induced expression of another ABC transporter ABCF4, plasma membrane ATPase and acetolactate synthase ALS1 were also observed (Table S4). FRD3 transports citrate into the xylem in *Arabidopsis* and over-expression of the gene in barley confers citrate efflux in the roots^11^. The H^+^-ATPase extrudes protons from cells to create a gradient of electrochemical proton across the plasma membrane. The electrochemical gradient promotes the activity of OA transporters and a passive efflux of organic anions from root tips^10^. Increasing evidence indicates a significant and positive correlation of plasma membrane H^+^-ATPase with the rates of citrate exudation under conditions of Al^3+^ stress and P-deficiency. Internally, a member of NRAMP family, Nrat1, was implicated in cooperating with ALS1 for Al^3+^ sequestration into vacuole in rice^3,4^, and internal Al^3+^ detoxification was mainly responsible for the 72 h Al^3+^ tolerance of TPRC2001–1 genotype in stylo^12^. Besides, STAR1, an ABC transporter in rice and *Arabidopsis*, form a complex with STAR2 to facilitate the export of UDP-glucose for cell wall modification and to mask Al^3+^-binding sites^4^. Thus, the expression patterns of above important transporters suggest that stylo harbors multiple strategies for Al^3+^-detoxification via Al^3+^ avoidance and tolerance mechanisms as well as modifications in the carbohydrate composition of the root cell wall.

Evidence indicates that Al^3+^ resistance in common bean depends on the capacity to sustain citrate synthesis for maintaining the cytosolic citrate pool enabling exudation^13^. Plants over-expressing genes related to citrate biosynthesis, such as CS and MDH, also showed more citrate production and efflux when compared to the untransformed control^2^. In an Al^3+^-toxicity environment, the substitution of Fe^3+^ by Al^3+^ in organisms leads to a block of TCA and oxidative phosphorylation due to dysfunctional proteins/enzymes that are dependent on Fe^3+^ to function^14^. In this study, expression of genes coding for Fe^3+^-dependent enzymes in TCA cycle, including ACO, IDH, SCS and SDH, were down-regulated (Fig. 3). Researchers documented a modified TCA cycle via oxidation of glyoxylate enabling the soil microbe *P. fluorescens* and rice exposed to Al^3+^ to generate ATP^15,16^. This novel ATP-producing module works in tandem with SCS, isocitrate lyase, acylating glyoxylate dehydrogenase and oxalate CoA-transferase to generate oxalate, a dicarboxylic acid involved in Al^3+^ immobilization^14^. By contrast, our data demonstrated no significant difference in expression for the above genes related to glyoxylate oxidation except down-regulation of SCS and up-regulation of CS. Given the decreased expression of MDH and increased expression of ACS, expression patterns of genes in TCA and in glyoxysome lead to an increased synthesis of citrate. The increased citrate anabolism is further supported by decreased expression of genes involved in the citrate-pyruvate cycle, glycolysis and beta oxidation. In consistence with these findings, intracellular citrate concentration increases with Al^3+^ dosage increasing in stylo roots as detected by our group previously^7^. Therefore, expression of genes involving citrate metabolism pathways has to be modulated to facilitate citrate synthesis for Al^3+^ detoxification in stylo roots.

OAs secretion from roots under Al^3+^ stress is achieved by the combined action of multiple transcription factors that regulate expression levels and tissue specificity^17^. Multiple transcription factors are required for STOP1/ART1-dependent secretion of malate in *Arabidopsis* and rice^17^. Similarly, our work revealed Al^3+^ activated expression of 2 transcription factor genes including STOP1 homolog (Table S6) and 3 STOP1-regulated genes including MATE and ALS1 (Table S7). In contrast to the finding in *Arabidopsis*^2^, the others of STOP1/ART1-regulated genes were not induced by Al^3+^. These results suggest that transcription regulation of citrate secretion differs from that of malate exudation.

According to the current reports, the signal cascades from Al^3+^ sensing to transcription activation remain to be resolved. To our knowledge, G-proteins relay the information from G-protein coupled receptors (GPCRs) on the plasma membrane by forming functional subunits of Gα and Gβγ^18^. Gα signaling is terminated by the intrinsic GTPase activity of Gα, in which ras-related small GTPase superfamily function as switch molecules by cycling between GTP-bound activated state to a GDP-bound inactivated state^19,20^. In consistence with our previous data in *Arabidopsis* and rye^9^, the involvement of G-proteins in Al^3+^ enhancement of citrate excretion in stylo roots was supported by pharmacological analysis and enhancement of GTPase activity revealed in this work. Importantly, our recent data demonstrated Al^3+^-induced secretion of oxalate in buckwheat and exudation of malate in wheat without elevation of GTPase activity in the roots of both species (unpublished data). Together with Al^3+^ decreased 4 of DEGs classed as ras family and G-proteins (Table S8), pharmacological observations point to a pivotal for heterotrimeric G-proteins specific in mediating Al^3+^-induced release of citrate at translation level.

It has been recognized that second messengers are important components in G-protein-mediated signaling pathways^18^. When a ligand binds to a GPCR, Gα can bind to and induce activity of PLC, which results in the cleavage of phosphatidylinositol 4, 5-bisphosphate (PIP_2_) into IP_3_ and DAG. IP_3_ then binds to its receptor and initiates intracellular calcium (as Ca^2+^) release from the ER. In agreement with previous findings in *Arabidopsis* and rye^9^, Al^3+^-enhancement of citrate secretion from stylo roots was significantly reduced by antagonists of PLC, IP_3_ receptor and DAG, whereas treatment with TSG, a specific activator of PLC, resulted in an increase in citrate release (Fig. S4). The expression patterns of unigenes participating in DAG and IP_3_ metabolism (Table S8), lend additional supports to functional involvement of PLC, IP_3_ and DAG in G-protein-medicated Al^3+^-signaling pathways also at translation level.

Ca^2+^ is another important component in G-protein-mediated signal in organisms^21^. In plants, Ca^2+^-decoding involves calcineurin B-like proteins (CBLs) that bind Ca^2+^ and activate CIPKs. The CBL-CIPK network targets to the cell membranes where CIPKs phosphorylate the substrate proteins and activate the transport activity^21^. In these processes, calcium-dependent protein kinases (CPKs) are activated by Ca^2+^ and lead to the phosphorylation of transcription factors, thereby regulating expression of responding genes including OA transporter genes^22^. Our previous data established cytoplasmic Ca^2+^ mediation of Al^3+^-induced citrate secretion in rye roots^23^. In this work, Al^3+^ disrupted expression of 21 Ca^2+^ signal members (Table S8). Although only 3 unigenes (At1g34300, FERONIA, PTP1) of DEGs were increased (Table S8), Al^3+^ has been reported to induce either increases or decreases in cytosolic Ca^2+^ depending on the experimental system studied^24^, and Al^3+^ triggers Ca^2+^ signaling activity at post-transcription, translation and posttranslational levels via protein phosphorylation and dephosphorylation as exemplified by recent reports^25,26^. Furthermore, the involvement of 16 up-regulated unigenes functional unkown (Table S10) in Ca^2+^ signal remains to be determined. Collectively, these results indicate that multiple Ca^2+^ signaling pathways function synergistically in resistance to Al^3+^ in stylo.

**Figure 5:**
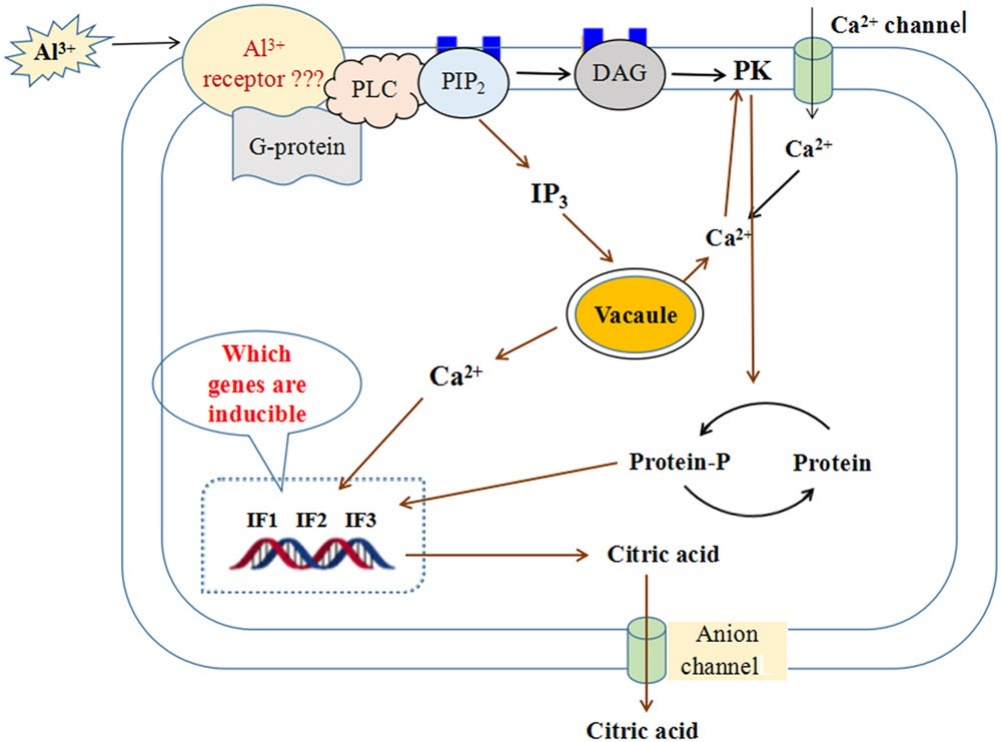
Putative model illustrating signaling cascades of Al^3+^- induced exudation of citrate from stylo roots. Al^3+^ interacts with a receptor on the plasma membrane to initiate a signaling pathway. Al^3+^ signal is transmitted via heterotrimeric G-proteins, inositol trisphosphate (IP_3_), diacylglycerol (DAG), Ca^2+^ and protein kinases (PK), leading to the activation of transcription and anion channels in plasma membrane, and resulting in citrate secretion from stylo roots.

In conclusion, Al^3+^ tolerance in stylo involves multiple strategies and enhancement of citrate anabolism. Importantly, the Al^3+^-signaling cascades compose G-proteins, PLC, IP_3_, DAG, Ca^2+^ and protein kinases, which lead to the activation of transcription and anion channels in plasma membrane, and result in root secretion of citrate (Fig. 5). However, the Al^3+^-receptors and the Al^3+^-activated genes responsible for citrate exudation remain to be determined.

## Methods

### Plant cultivation and measurements of Al^3+^ treatments

R2 seeds were obtained from Yunnan Academy of Agricultural Sciences (Kunming city, Yunnan province, Southwest China). Cultivation of seeds was performed according to Li *et al*.^7^. Seedlings after growing in the dark for 4 d at 25 °C were grown on a floating mesh in plastic containers with full nutrient solution. The nutrient solution described by Li *et al*.^7^ was renewed every other day. For Al^3+^ treatment, the 30-day-old uniform seedlings were used. Seedlings pre-grown overnight in a 0.5-mM CaCl_2_ solution (pH 4.3) were transferred into a 0.5-mM CaCl_2_ solution containing 0 (control), 5, 15, 25, 50 and 100 µM AlCl_3_ (pH 4.3) for 24 h, respectively. Each experiment was repeated for three times. RRG and Chrome Azurol S staining methods were used to measure the effects of Al^3+^ treatment, and citrate exudation was determined using HPLC as described previously^7^.

### RNA extraction, library construction and sequencing

R2 seedings cultivated as above were subject to treatments of T01, T02 and T03 with three replicates for each treatment and 30 seedlings for each replicate. After 2, 6 and 24 h, root apex (0–1 cm) of 10 seedlings chosen randomly from the same treatment were cut and collected into centrifuge tube, and frozen in liquid nitrogen immediately. Total RNA was extracted using an RNeasy Plant Mini Kit (QIAGEN) and was digested with DNase I (TAKARA). RNA purity, concentration and integrity were checked using Nanodrop 1000 (Thermo Fisher Scientific, CA, USA), Qubit® 2.0 (Life Technologies, CA, USA) and Aglient 2100 (Aglient Technologies, CA, USA), respectively. The quality of the total RNA ranged from RIN 9.6 to 9.9 over the suggested RIN 8, proving to be excellent and stable for RNA-Seq analysis. Total RNA from the three time points of the same treatment were pooled, and a total amount of 3 μg RNA was used mRNA sample preparations. Ploy(A) mRNAs were purified separately from the 3 RNA pools using Oligo (dT), and fragmented. The first strand cDNA was reverse transcribed with random hexamers, followed by second strand cDNAs synthesis with DNA polymerase I (New England BioLabs) and RNase H (Invitrogen). After purification using AMPure XP beads (New England Biolabs), adaptor-ligation and size-selection, the double-stranded cDNA was subjected to PCR-enrichment for 14–16 cycles. The quality of the libraries was checked using Qubit2.0, Agilent 2100 Bioanalyzer and Q-PCR. The cDNA library was subject to paired-end sequencing in HiSeq X Ten system with PE125 by Biomarker Technologies (Beijing, China). The raw sequence data obtained have been deposited at the NCBI in the Short Read Archive (SRA) database under the accession number SRP131721.

### Transcriptome assembly and annotation

Raw reads were quality-checked, and adaptor sequences and low quality reads were removed according to the method of Chen *et al*.^10^. The obtained clean reads were assembled into unigenes using the Trinity program^27^, which partitions the sequence data into many individual de Bruijn graphs, and processes each graph independently to extract full-length splicing isoforms and to tease apart transcripts derived from paralogous genes. For annotation, all unigenes, which proved to be over 200 bp, were subjected to BLAST search (E-value < 1e-5) against the databases of NCBI non-redundant protein (NR), Swissprot, GO, KOG, eggNOG, KEGG and Pfam.

### Differential expression and enrichment analyses

The clean reads per sample were mapped back to assembled contigs by Bowtie^28^. Transcripts abundance was calculated and represented by reads per kilobase of transcript per million mapped reads (RPKM) value using the RSEM package^29^. The assembled transcripts with RPKM ≥ 0.1 were defined as expression.

The assembled transcripts with more than 10 reads mapped were subjected to differential expression analysis between treatments by DESeq^30^. DEGs were defined when FDR (false discovery rate) < 0.001 and log_2_FC > 2, and were extracted for GO and KEGG enrichment analysis, which was tested using Fisher’s exact test at a significance cutoff of FDR < 0.05.

### Real-time RT-PCR

Total RNA was isloated from R2 seedlings for the RNA-Seq library construction. After DNase I treatment, the RNA samples were subjected to the first-strand cDNA synthesis using HiScript® 1st Strand cDNA Synthesis Kit (Vazyme). One twentieth of the cDNA products and the SYBR® Green Master Mix kit (Vazyme) were used for qRT-PCR analysis with the CFX96 Real-Time PCR Detection System (Bio-Rad) with primers listed in Table S9. Reactions were performed in triplicate for each sample with SgEF-1a as an endogenous control. Relative expression was calculated with the 2^−∆∆Ct^ method.

### GTPase activity and pharmacological assays

R2 seedlings aforementioned were exposed to a 0.5-mM CaCl_2_ solution (pH4.3) containing 50 μM AlCl_3_ (+Al^3+^) or not (−Al^3+^) for 3 h. The details for isolation of plasma membrane by two-phase partitioning protocol (6.2% dextran T-500 and 6.2% polyethylene glycol 3350), measurement of GTPase activity with a spectrofluorometer (LS-55, Perkin Eimer Inc., Liantrisant, UK) and pharmacological analyses referred to Li *et al*.^7^.

### Data availability

Raw sequence reads can be found in the SRA database under BioProject PRJNA431518. This Transcriptome Assembly project has been deposited SRA database under the accession SRP131721.

## Electronic supplementary material

Supplementary Information
