# Supplementary material for: Insights into aluminum-tolerance pathways in Stylosanthes as revealed by RNA-Seq analysis
Source: Sci Rep. 2018 Apr 17;8:6072. doi: 10.1038/s41598-018-24536-3 (PMC5904178; doi:10.1038/s41598-018-24536-3)
Supplement: Supplementary file 1 — Supplementary Information [file 41598_2018_24536_MOESM1_ESM.pdf]

Supplementary Information:

Insights into aluminum-tolerance pathways in *Stylosanthes* as revealed by RNA-Seq analysis

Authors:

Caode Jiang[1], Lusheng Liu[1], Xiaofeng Li[2], Rongrong Han[1], Yunmin Wei[1] & Yongxiong Yu[1,*]

Author affiliations:

[1]Forage Laboratory, College of Animal Science and Technology, Southwest University, Chongqing, 400715, China

[2]College of Agriculture, Guangxi University, Nanning, 530005, China

Authors for correspondence:

Caode Jiang (jcdpjx@swu.edu.cn), Yongxiong Yu (email: yuyongxiong8@126.com)

1. Supplementary Figures

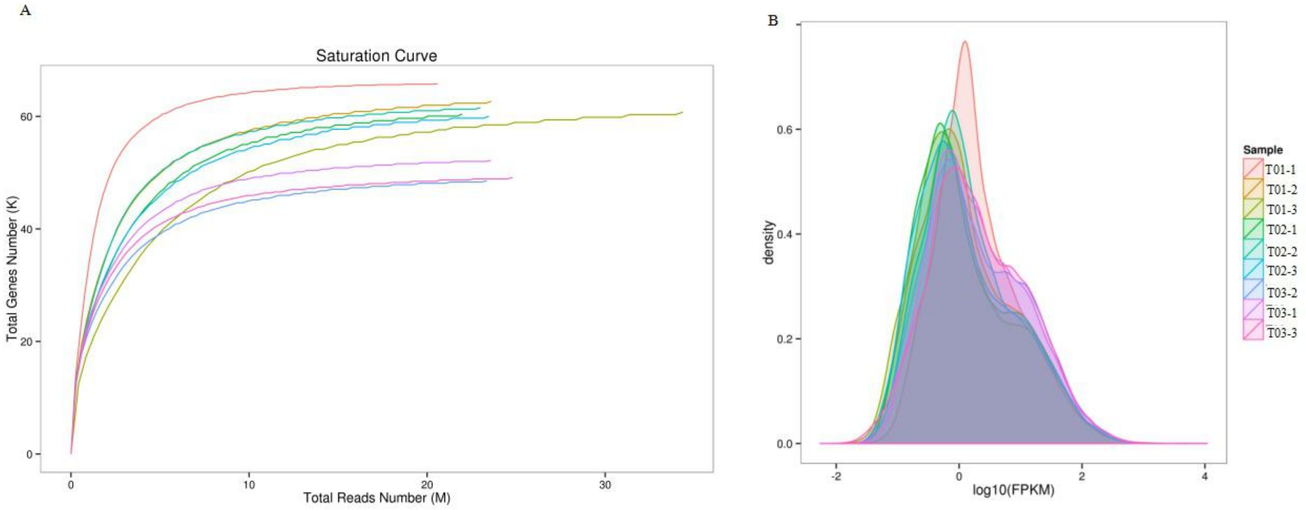

Fig. S1 Saturation simulation diagram of transcriptomic data (A) and comparison chart of density distribution of FPKM for each sample (B).

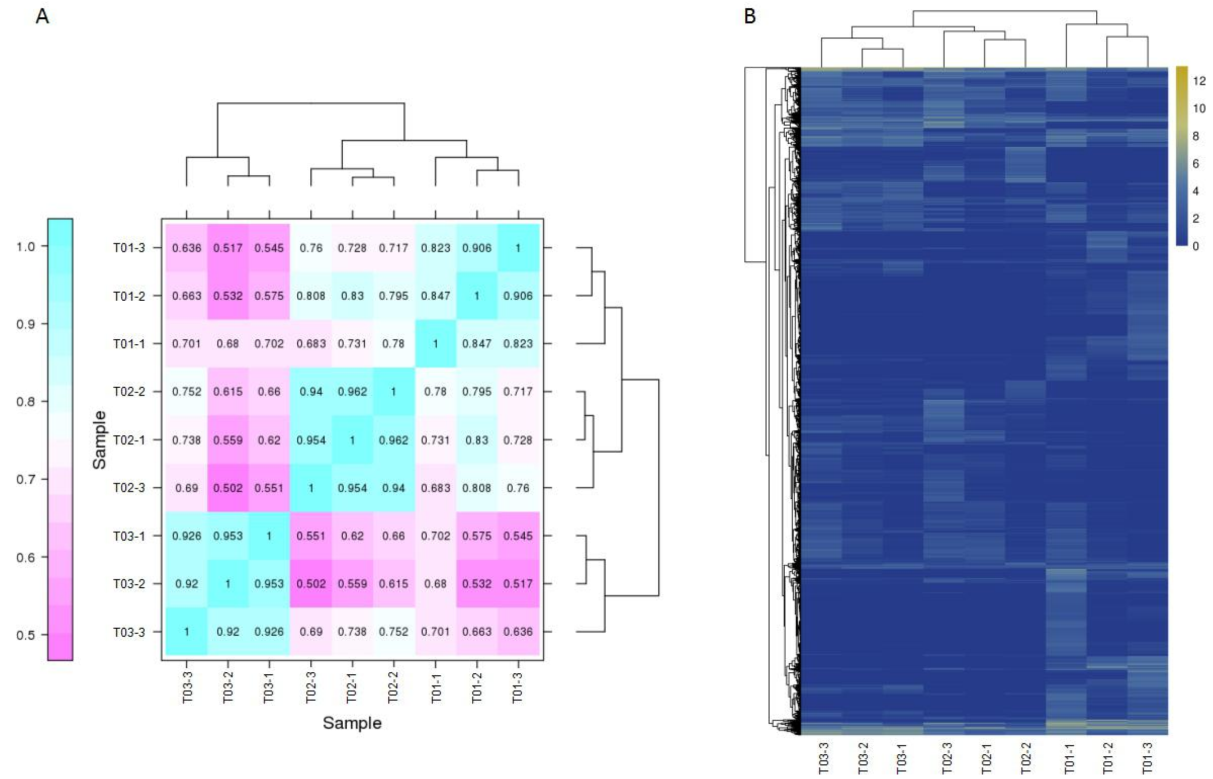

Fig. S2 Correction of all gene expression based on determination coefficients (R^2) (A) and cluster of differentially expressed genes between the 9 samples (B). Gene expression values are log10 transformed.

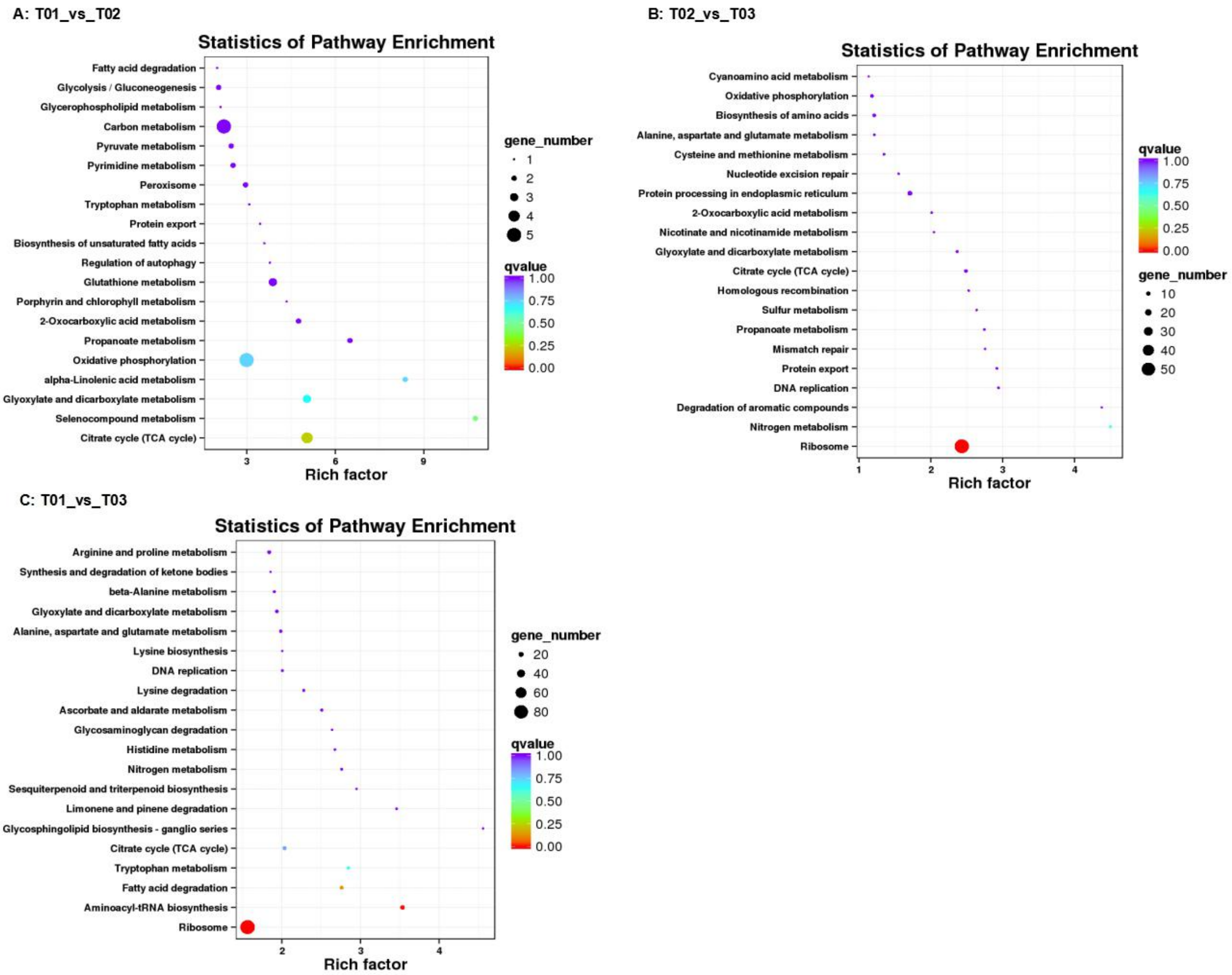

Fig. S3 KEGG enrichment of differential expressed unigenes. (A) The acid treatment vs the control, (B) The Al^{3+} treatment vs the control; (C) The Al^{3+} treatment vs the acid treatment.

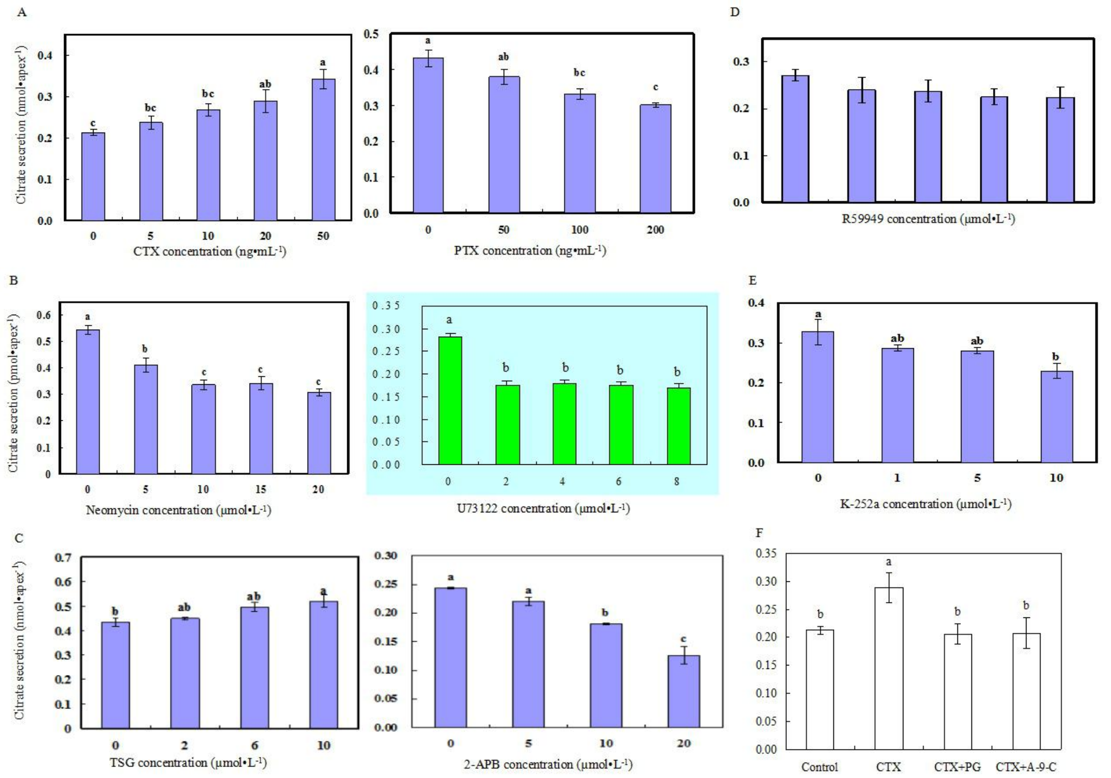

Fig. S4 Pharmacological analysis of effects of G-protein pathways on Al^{3+}-induced secretion of citrate in R2 roots. (A), the G-protein agonist

cholera toxin (CTX) and antagonist pertussis toxin (PTX); (B), phospholipase C antagonist neomycin and U73122; (C), inositol trisphosphate receptor agonist thapsigargin (TSG) and antagonist 2-aminoeyhyl diphenylborinate (2-APB); (D), diacylglycerol specific inhibitor R599499; (E), protein kinase specific inhibitor K-252a; and F) anion channel inhibitors phenylglyoxal (PG) and anthracene-9-carboxylic acid (A-9-C, 30 μmol·L^{-1}) on CTX-triggered (20 ng·mL^{-1}) citrate efflux. R2 seedlings were exposed to 50 μmol/L $AlCl_3$ for 3 h. Each value is expressed as mean ± SEM (n = 3 replicates). Different letters above the columns indicate the significance at *P* < 0.05, according to Tukey's Multiple Range Test.

2. Supplementary Tables

Table S1 The length distribution of assembled transcripts and unigenes

Length Range	Transcript	Unigene
200-300	11,272 (8.61%)	8,844 (10.63%)
300-500	26,929 (20.57%)	19,784 (23.78%)
500-1000	42,200 (32.23%)	29,115 (35.00%)
1000-2000	31,047 (23.71%)	17,259 (20.74%)
2000+	19,485 (14.88%)	8,195 (9.85%)
Total Number	130,933	83,197
Total Length	148,799,476	79,796,360
N50 Length	1,664	1,307
Mean Length	1,136.46	959.13

Table S2 Summary of annotation for unigenes

#Anno_Database	Annotated No.	300 ≤ length < 1000	Length ≥ 1000
COG_Annotation	21,089	10,713	9,196
GO_Annotation	19,383	9,431	8,671
KEGG_Annotation	23,135	12,537	9,062
KOG_Annotation	34,594	17,946	14,608
Pfam_Annotation	42,822	21,146	19,668
Swissprot_Annotation	32,597	15,642	15,126
eggNOG_Annotation	49,963	25,355	21,554
nr_Annotation	50,173	25,231	21,774
All_Annotated	54,976	28,636	22,723

Table S3 Top 10 GO enrichment in differential expressed unigenes

T01_vs_T02	KS	T02_vs_T03	KS	T02_vs_T03	KS
**Biologocal process (BP)**					
translation	1.40E-13	translation	2.30E-13	translation	6.50E-14
regulation of transcription, DNA-templated	5.50E-5	regulation of transcription, DNA-templated	4.90E-05	regulation of transcription, DNA-templated	0.00012
small GTPase mediated signal transduction	0.000140	small GTPase mediated signal transduction	0.00014	small GTPase mediated signal transduction	0.00019
ornithine metabolic process	0.00017	ornithine metabolic process	0.00017	ornithine metabolic process	0.0002

response to karrikin	0.00022	response to karrikin	0.00021	response to karrikin	0.00027
plant-type cell wall modification	0.00053	plant-type cell wall modification	0.00052	plant-type cell wall modification	0.00066
fatty acid biosynthetic process	0.0009	fatty acid biosynthetic process	0.00081	regulation of response to biotic stimulus	0.00113
organ morphogenesis	0.0011	gravitropism	0.00161	regulation of response to external stimus	0.00124
regulation of stomatal movement	0.0017	regulation of stomatal movement	0.00165	xylan metabolic process	0.00131
gravitropism	0.00174	organ morphogenesis	0.00169	fatty acid biosynthetic process	0.0015
**Cellular component (CC)**					
ribosome	7.00E-19	ribosome	1.30E-18	ribosome	8.50E-19
small ribosomal subunit	9.30E-06	small ribosomal subunit	1.20E-05	small ribosomal subunit	1.10E-05
ribonucleoprotein complex	0.00016	ribonucleoprotein complex	0.00014	ribonucleoprotein complex	0.00026
trans-Golgi network	0.00057	trans-Golgi network	0.00048	cytosolic ribosome	0.0006
cytosolic ribosome	0.00075	cytosolic ribosome	0.00061	trans-Golgi network	0.00078
cytosolic large ribosomal subunit	0.00452	plant-type cell wall	0.00664	cell periphery	0.00274
nucleolus	0.00615	cytosolic large ribosomal subunit	0.0073	nucleolus	0.00548
plant-type cell wall	0.00764	nucleolus	0.00795	cytosolic large ribosomal subunit	0.00563
cell periphery	0.01382	cell periphery	0.01297	plant-type cell wall	0.01018
MCM complex	0.02005	MCM complex	0.01921	organelle membrane	0.01446
**Molecular function (MF)**					
structural constituent of ribosome	< 1e-30	structural constituent of ribosome	< 1e-30	structural constituent of ribosome	3.00E-30
beta-galactosidase activity	6.50E-06	beta-galactosidase activity	6.20E-06	beta-galactosidase activity	8.90E-06
phospholipase activity	1.90E-05	phospholipase activity	1.70E-05	phospholipase activity	2.70E-05
carbohydrate binding	0.0002	carbohydrate binding	0.00017	carbohydrate binding	0.00037
sequence-specific DNA binding transcripion	0.00047	sequence-specific DNA binding transcription	0.00042	sequence-specific DNA binding transcription	0.00089
pectinesterase activity	0.001	pectinesterase activity	0.00095	pectinesterase activity	0.00132
transferase activity, transferring hexosyl group	0.00124	transferase activity, transferring hexosyl group	0.00107	histone binding	0.00143
histone binding	0.00126	histone binding	0.00123	phosphoric diester hydrolase activity	0.00192
phosphoric diester hydrolase activity	0.0014	phosphoric diester hydrolase activity	0.00131	aspartyl esterase activity	0.00243
aspartyl esterase activity	0.0019	protein serine/threonine kinase activity	0.00167	transition metal ion binding	0.00253

Table S4 Al^{3+}-induced transporter genes in R2 roots

Gene ID	Description	Log_2 (fold change)			Category
		T01_vs_T02	T02_vs_T03	T01_vs_T03	
c329012.graph_c0	ABC transporter G family member 25 (ABCG25)	N.S.	-3.05	-3.90	ABC family
c333559.graph_c1	ABC transporter C family member 14 (ABCC14)	-3.21	N.S.	-4.55	
c333797.graph_c0	ABC transporter G family member 22 (ABCG22)	N.S.	-4.96	-5.53	
c340676.graph_c1	ABC transporter F family member 4 (ABCF4)	N.S.	N.S.	2.69	
c341230.graph_c0	ABC transporter C family member 7 (ABCC7)	-5.57	N.S.	-5.70	

c331542.graph_c0	Solute carrier family 35 member G1 (SLC35G1)	N.S.	2.72	5.55	MATE family
c329070.graph_c0	MATE efflux family protein	2.86	N.S.	2.98	
c333945.graph_c0	Sugar carrier protein C (STC)	N.S.	2.59	6.15	Major Facilitator Superfamily
c334178.graph_c0	Sugar transport protein 2 (STP2)	N.S.	N.S.	-6.11	
c341187.graph_c0	Sugar transport protein 11 (STP11)	-4.46	3.18	N.S.	
c341366.graph_c0	Hypothetical protein BATDEDRAFT_89188	N.S.	5.22	4.93	
c338283.graph_c1	Hypothetical protein PHYSODRAFT_523004	N.S.	3.15	2.89	
c341366.graph_c0	Hypothetical protein BATDEDRAFT_89188	N.S.	5.22	4.93	
c303083.graph_c0	Mitochondrial phosphate carrier protein 3, mitochondrial (MPCP3)	-3.27	N.S.	-5.98	Inorganic ion transporter
c320542.graph_c0	Calcium-transporting ATPase 2, endoplasmic reticulum-type (ECA2)	N.S.	N.S.	-4.46	
c327929.graph_c0		N.S.	N.S.	-4.61	
c332838.graph_c0	Ammonium Transporter Family	N.S.	N.S.	-3.98	
c346112.graph_c0	Solute carrier family 40 member 2 (S40A2)	N.S.	N.S.	3.15	
c341390.graph_c0	Ferric reduction oxidase 4 (FRO4)	N.S.	4.29	N.S.	
c339453.graph_c0	Pyrophosphate-energized vacuolar membrane proton pump	N.S.	-5.80	-5.30	H^+-ATPase
c339259.graph_c0		-2.90	-5.35	-8.27	
c339453.graph_c3		N.S.	N.S.	-6.26	
c341455.graph_c0	Plasma membrane ATPase (Os04g0656100)	N.S.	3.08	N.S.	
c216702.graph_c0	V-type proton ATPase 16 kDa proteolipid subunit	N.S.	N.S.	-5.81	
c339055.graph_c1	V-type proton ATPase catalytic subunit A (VPH1)	N.S.	-2.83	-5.60	
c331477.graph_c0	Choline transporter like family	-3.73	N.S.	-7.28	Plasma-membrane choline transporter
c333635.graph_c0	Choline transporter-like domain-containing protein	N.S.	N.S.	-3.15	
c335074.graph_c0	Hypothetical protein H257_00933	N.S.	-4.07	-5.74	
c339130.graph_c0	Hypothetical protein PHYSODRAFT_293340	N.S.	3.05	3.39	
c340447.graph_c1	Probable folate-biopterin transporter 4 (FBT4)	-3.26	N.S.	-3.56	BT1 family
c340819.graph_c0	Probable folate-biopterin transporter 5 (At5g25040)	-6.18	N.S.	-4.76	
c340762.graph_c0	Hypothetical protein, variant [Phytophthora parasitica INRA-310]	N.S.	4.95	N.S.	Lipid transport and metabolism
c340823.graph_c1	Acetolactate synthase (ALS1/ STAR1)	N.S.	2.26	3.89	
c302155.graph_c0	ADP/ATP carrier protein 3, mitochondrial (AAC3)	-3.96	N.S.	-7.44	Others
c337471.graph_c0	Hypothetical protein F443_12489	N.S.	3.09	N.S.	
c340718.graph_c0	Hypothetical protein PHYSODRAFT_556902	N.S.	4.07	N.S.	

N.S., not significant.

Table S5 Expression change of genes involved in citrate metabolism

No.	Gene name	GeneID	Log$_2$(fold change)		
			T01_vs_T02	T02_vs_T03	T01_vs_T03
1	Citrate synthase (CS)	c309470.graph_c0	N.S.	5.64	5.63
2	Aconitate hydratase (ACO)	c335652.graph_c0	N.S.	N.S.	-4.02
		c335652.graph_c1	-2.79	N.S.	-6.32
		c338461.graph_c0	-2.50	-5.26	-8.19
3	Isocitrate dehydrogenase (IDH)	c329683.graph_c1	-3.29	N.S.	-5.45
4	2-oxoglutarate dehydrogenase (OGDH)	c328278.graph_c0	N.S.	-3.42	-6.29
5	Succinyl-CoA synthetase (SCS)	c318464.graph_c0	-2.10	-5.77	-7.89
		c316673.graph_c0	-2.19	N.S.	-7.47
6	Succinate dehydrogenase (SDH)	c328969.graph_c0	N.S.	N.S.	-5.18
		c329006.graph_c0	N.S.	N.S.	-5.99
7	Phosphoenolpyruvate carboxykinase (PPCK)	c330658.graph_c0	N.S.	-5.08	-6.58
8	Malate dehydrogenase, cytoplasmic (MDH)	c319636.graph_c0	-3.50	N.S.	-7.05
9	Pyruvate dehydrogenase (PDH)	c328301.graph_c0	N.S.	N.S.	-6.14
10	Acetyl-coenzyme A synthetase, chloroplastic/glyoxysomal (ACS)	c341131.graph_c1	N.S.	3.32	N.S.
11	Fructose-bisphosphate aldolase (ALDO)	c323548.graph_c0	N.S.	N.S.	-4.89
12	Glyceraldehyde-3-phosphate dehydrogenase (GAPDH)	c318236.graph_c0	N.S.	N.S.	-5.31
13	Triacylglycerol lipase (TGL)	c313221.graph_c0	N.S.	-5.35	-5.37
14	Acyl-CoA dehydrogenase (ACAD)	c294674.graph_c0	N.S.	N.S.	-5.61
		c309154.graph_c0	N.S.	N.S.	-5.24
		c314698.graph_c0	N.S.	N.S.	-5.44
15	Probable trans-2-enoyl-CoA reductase, mitochondrial (TER)	c302431.graph_c0	N.S.	N.S.	-5.97
16	Enoyl-CoA hydratase (ECH)	c321370.graph_c0	N.S.	N.S.	-5.75
17	3-hydroxyacyl-CoA dehydrogenase (HAD)	c318974.graph_c0	N.S.	N.S.	-6.03
18	3-ketoacyl-CoA thiolase 2 (KAT2)	c319716.graph_c0	N.S.	-4.26	-4.68

Table S6 Transcriptional changes of genes involved in transcription and translation under Al^{3+} treatment

#ID	Description	Log$_2$(Fold change)		
		T01_vs_T02	T02_vs_T03	T01_vs_T03
**Transcription**				
c291986.graph_c3	Protein SENSITIVE TO PROTON RHIZOTOXICITY 1(STOP1)	N.S.	N.S.	2.91
c333017.graph_c2	Hypothetical protein SDRG_02326 (Zinc finger, ZZ type)	N.S.	2.89	N.S.
c244648.graph_c0	Transcription factor BTF3 homolog 4-like	N.S.	N.S.	-5.50
c275543.graph_c0	Transcription factor TFIIIB	N.S.	N.S.	-4.68
c297335.graph_c0	Heat stress transcription factor A-2a	N.S.	N.S.	-6.27
c308365.graph_c0	Transcription factor MYB57	-4.75	N.S.	-7.16
c341161.graph_c0	Uncharacterized zinc finger protein At4g06634	N.S.	N.S.	-6.19

c323805.graph_c0	Transcription factor RAX2	N.S.	N.S.	-3.75
c329116.graph_c0	Transcription factor MYB98	N.S.	N.S.	-4.42
c330265.graph_c0		N.S.	N.S.	-5.61
c318963.graph_c0	Probable elongation factor 1-gamma 2	-2.60	N.S.	-6.93
c315837.graph_c0	Probable mediator of RNA polymerase II transcription subunit 36b	N.S.	N.S.	-6.75
c329304.graph_c0	RuvB-like protein 1	N.S.	N.S.	-4.14
c332380.graph_c0	Splicing factor U2af large subunit A	N.S.	N.S.	-5.70
c336098.graph_c0	Glycine-rich RNA-binding protein 2, mitochondrial	-3.51	N.S.	-6.77
c338944.graph_c0	Intraflagellar transport protein 172	N.S.	-5.20	-7.69
**Translation and posttranslational modification**				
c247351.graph_c1	40S ribosomal protein S30 (RPS30C)	N.S.	N.S.	3.74
c332492.graph_c0	Hypothetical protein EMIHUDRAFT_362303	N.S.	N.S.	5.99
c335676.graph_c1	Hypersensitive-induced response protein HIR3	N.S.	4.62	8.58
c335919.graph_c0	Aspartic proteinase A2 (APA2)	N.S.	N.S.	3.27

Table S7 Induction of STOP1 regulated genes by Al^{3+} in R2 roots

RAP ID	Description	Gene ID	Log$_2$(fold change)		
			T01_vs_T02	T02_vs_T03	T01_vs_T03
Os01g0860500	Chitinase	c315389.graph_c0	-2.71	N.S.	-5.29
Os10g0206800	Multidrug and toxic compound extrusion (MATE) family protein	c325940.graph_c1	N.S.	N.S.	-6.01
		c329070.graph_c0	2.86	N.S.	2.98
Os09g0479900	Subtilisin-like Ser protease SBT5.4	c289101.graph_c0	-5.86	N.S	-2.99
Os03g0755100	At ALS1 homolog	c332092.graph_c0	N.S	2.45	2.76
Os04g0494900	Unknown function DUF642 family	c133280.graph_c0	N.S	N.S.	4.15

Table S8 Transcription changes of signal transduction genes by Al^{3+}

#ID	Description	log2(fold change)			Category
		T01_vs_T02	T02_vs_T03	T01_vs_T03	
c279616.graph_c0	Ras-related protein RIC1	N.S.	N.S.	-3.62	Ras family and G-protein
c310836.graph_c0	ADP-ribosylation factor 1	N.S.	-2.73	-4.94	
c323583.graph_c0	Guanine nucleotide-binding protein subunit beta-like protein	-3.77	N.S.	-6.83	
c340198.graph_c0	G-protein beta subunit	N.S.	N.S.	-4.75	
c291165.graph_c0	Putative phosphatidylglycerol/phosphatidylinositol transfer protein DDB_G0282179	N.S.	N.S.	-6.11	Diacylglycerol and inositol phosphate metabolism
c186435.graph_c0	Putative cdp-diacylglycerol-inositol 3-phosphatidyltransferase	N.S.	N.S.	-5.44	
c334990.graph_c1	Inositol-1,4,5-triphosphate receptor	N.S.	N.S.	-5.70	Ca^{2+} binding protein
c127005.graph_c0	Probable calcium-binding protein CML13	N.S.	N.S.	-5.73	
c135211.graph_c0	Putative calmodulin	-2.53	N.S.	-6.16	
c308290.graph_c0	Caltractin	N.S.	N.S.	-5.81	
c327262.graph_c0	CBL-interacting protein kinase 30	N.S.	N.S.	-4.23	CBL-interacting

c328302.graph_c0	CBL-interacting serine/threonine-protein kinase 23	N.S.	N.S.	-3.27	protein kinase
c319697.graph_c0	CBL-interacting protein kinase 28	N.S.	N.S.	-5.36	
c328408.graph_c0	Probable serine/threonine-protein kinase CCRP1	N.S.	N.S.	-6.29	Serine/threonine protein kinase
c330877.graph_c0	Serine/threonine-protein kinase AtPK1/AtPK6	N.S.	N.S.	-3.95	
c334595.graph_c1	Probable inactive cyclic nucleotide-dependent protein kinase At2g20050	N.S.	N.S.	-6.62	
c336746.graph_c1	Serine/threonine-protein kinase TIO	N.S.	N.S.	-5.13	
c305058.graph_c0	G-type lectin S-receptor-like serine/threonine-protein kinase At1g34300	N.S.	N.S.	3.09	
c323334.graph_c0	Casein kinase I isoform delta-like	N.S.	N.S.	-3.72	
c320249.graph_c0	SNF1-related protein kinase catalytic subunit alpha KIN10	N.S.	N.S.	-5.72	
c336770.graph_c0		N.S.	N.S.	-4.99	
c128789.graph_c0	Receptor-like protein kinase FERONIA	N.S.	N.S.	2.74	
c315824.graph_c0	Casein kinase I isoform delta-like	N.S.	N.S.	-5.96	
c339764.graph_c1	Hypothetical protein F441_22463 [Phytophthora parasitica CJ01A1]	N.S.	N.S.	6.07	Carboxylesterase family
c296709.graph_c0	Probable purple acid phosphatase 20 (Precursor)	N.S.	5.31	4.24	
c296709.graph_c1		N.S.	N.S.	4.58	
c300233.graph_c0	Serine/threonine-protein phosphatase PP2A catalytic subunit	N.S.	N.S.	-5.86	Phosphatase
c332173.graph_c0	Protein phosphatase 2A regulatory B subunit	N.S.	N.S.	-5.18	
c335813.graph_c0	Protein-tyrosine-phosphatase PTP1	N.S.	4.60	N.S.	
c332173.graph_c0	Protein phosphatase 2A regulatory B subunit	N.S.	-2.40	-4.11	
c313664.graph_c0	14-3-3-like protein GF14 iota	-2.81	N.S.	-5.05	Others
c325152.graph_c0	Hypothetical protein GUITHDRAFT_140475	N.S.	N.S.	-4.68	
c328335.graph_c1	Cell division cycle protein 48 homolog	N.S.	-3.87	-4.85	
c326263.graph_c0	Hypothetical protein PHYSODRAFT_305946	N.S.	N.S.	-3.33	
c332084.graph_c0	Hypothetical protein GUITHDRAFT_118521	N.S.	N.S.	-3.91	
c334019.graph_c0	Hypothetical protein, variant [Saprolegnia diclina VS20]	N.S.	-3.87	-4.91	

Table S9 Primers for qRT-PCR

Gene ID	Gene description	Primer sequence (5'-3')	Annealing (°C)	Size (bp)
c340332.graph_c0	Phosphatidylinositol-4-phosphate 5-Kinase (PIPK5D)	F: GCTTCATCTCGAGTGTGCTG<br>R: GCCAGGCTCGTAATCAACAG	55	189
c336618.graph_c0	G-protein alpha-2 subunit (GPA2)	F:GATGATCGGTGTGCAGTGTC<br>R:CGATGCCAAGAACGAAGAGG	56	163
c344276.graph_c0	Phosphoinositide phospholipase C (PLC)	F:TGTTCCACCTCAAGACCTCA<br>R:TCCTTCTCCGGCCTTTTCAT	57	177
c321767.graph_c0	Diglyceride acyltransferase (DGAT)	F:ACACCATCAACACCAAACCG<br>R:ACCCTCGCGTGGATATTTCT	57	213
c291986.graph_c3	Zinc finger protein STOP1 homolog	F:AGCAGCCATGGAAGTTGTTG<br>R:TCCAGGAACCGAGGATTTGG	58	244

c341388.graph_c5	ABC transporter C family member 9 (ABCC9)	F:GCTCACCGACACTGAAGTTC<br>R:GTGCGTGCCAAGATCAGTAT	58	196
	SgEF-1a	F:TGGGAGGTATTGACAAGCGT<br>R:CGGGAGCATCAATGACAGTG	59	189

Table S10 Up-regulation of other genes function unknown by Al^{3+} in R2 roots

Gene ID	Description	Log_2(fold change)		
		T01_vs_T02	T02_vs_T03	T01_vs_T03
c315372.graph_c0	PREDICTED: protein FAR1-RELATED SEQUENCE 5-like	N.S.	3.34	N.S.
c212720.graph_c0	BURP domain-containing protein 3	N.S.	N.S.	5.54
c310871.graph_c1	Elicitin-like protein	N.S.	N.S.	7.80
c128916.graph_c0	Hypothetical protein PHAVU_009G043300g	N.S.	N.S.	4.13
c319837.graph_c0	Hypothetical protein PPTG_01892	N.S.	4.11	2.42
c330224.graph_c0	Hypothetical protein L915_06175	N.S.	8.20	5.31
c330364.graph_c0	Hypothetical protein PHYSODRAFT_354683	N.S.	6.38	N.S.
c332301.graph_c0	Hypothetical protein PHYSODRAFT_285292	N.S.	5.44	N.S.
c332927.graph_c3	Hypothetical protein PHYSODRAFT_527875, partial	N.S.	5.33	5.32
c324323.graph_c0	Hypothetical protein L484_018823	N.S.	6.67	N.S.
c334725.graph_c0	Conserved hypothetical protein [Phytophthora infestans T30-4]	N.S.	2.95	N.S.
c336886.graph_c0		N.S.	4.31	N.S.
c336855.graph_c0	Hypothetical protein L917_16820	N.S.	3.14	4.39
c336863.graph_c0	Hypothetical protein L917_10077	N.S.	7.08	3.17
c337256.graph_c0	Hypothetical protein PPTG_13010	N.S.	3.50	N.S.
c337521.graph_c0	Hypothetical protein PHYSODRAFT_284994	-2.59	2.91	N.S.
c339954.graph_c0	Hypothetical protein PHYSODRAFT_355861	N.S.	2.40	N.S.
c340765.graph_c0	Hypothetical protein L916_01294	N.S.	2.57	N.S.
c340796.graph_c0	Hypothetical protein PHYSODRAFT_523306	N.S.	3.16	N.S.
c340985.graph_c0	Hypothetical protein F444_10199	N.S.	3.36	3.95
c341362.graph_c0	Hypothetical protein L915_03342	N.S.	4.85	N.S.
c344744.graph_c0	--	N.S.	3.47	3.60
c290106.graph_c0	--	N.S.	5.53	5.24
c129285.graph_c0	--	N.S.	N.S.	4.52
c318527.graph_c0	--	N.S.	4.82	N.S.
c311094.graph_c0	--	N.S.	4.11	8.40
